# Vaccine-induced Immunity Circumvented by Typical *Mycobacterium tuberculosis* Beijing Strains

**DOI:** 10.3201/eid1502.080795

**Published:** 2009-02

**Authors:** Kristin Kremer, Marieke J. van der Werf, Betty K.Y. Au, Dang D. Anh, Kai M. Kam, H. Rogier van Doorn, Martien W. Borgdorff, Dick van Soolingen

**Affiliations:** National Institute for Public Health and the Environment, Bilthoven, the Netherlands (K. Kremer, B.K.Y. Au, D. van Soolingen); KNCV Tuberculosis Foundation, The Hague, the Netherlands (M.J. van der Werf, M.W. Borgdorff); National Institute of Hygiene and Epidemiology, Hanoi, Vietnam (D.D. Anh); Department of Health, Hong Kong Special Administrative Region, People’s Republic of China (K.M. Kam); University of Amsterdam, Amsterdam, the Netherlands (H.R. van Doorn, M.W. Borgdorff)

**Keywords:** *Mycobacterium tuberculosis*, Beijing strain, vaccine escape and drug resistance, W strain, BCG vaccination, drug resistance, MDR, dispatch

## Abstract

The frequency of typical and atypical Beijing strains of *Mycobacterium tuberculosis* was determined in the Netherlands; Vietnam; and Hong Kong Special Administrative Region, People’s Republic of China. The strains’ associations with drug resistance, *M. bovis* BCG vaccination, and patient characteristics were assessed. BCG vaccination may have positively selected the prevalent typical Beijing strains.

*Mycobacterium tuberculosis* Beijing strains cause a substantial proportion of tuberculosis (TB) cases worldwide (*1*). Experiments in a BALB/c mouse model (*2*) and a rabbit model (*3*) supported the hypothesis that Beijing strains might represent “escape variants” of *M. bovis* BCG vaccination (*4*). In a study in Ho Chi Minh City, Vietnam, presence of a BCG scar correlated, but not significantly, with infection by Beijing strains (*5*).

The Beijing clade is highly prevalent in Asia, where the proportion of TB cases caused by strains of this clade usually is stable over time, and no association with drug resistance has been recorded. In other areas (e.g., Cuba, South Africa, countries of the former Soviet Union, and Vietnam), Beijing strains are emerging and associated with resistance to anti-TB drugs (*1*). The Beijing clade comprises at least 2 major subgroups, which share the characteristic spoligotype pattern (*6**–**8*): typical and atypical Beijing strains. Typical (“modern” [*8**,**9*]) Beijing strains, including W strains (*7*), exhibit highly similar, multicopy insertion sequence (IS) *6110* restriction fragment length polymorphism (RFLP) patterns and have alterations in putative mutator genes (*4**,**10*). Atypical (“ancestral” [*8**,**9*]) Beijing strains more closely resemble the common ancestor of the Beijing clade (*6**–**8**,**10*). The ability of these Beijing clade subgroups to gain resistance or circumvent BCG vaccine–induced immunity may differ and thus explain the differences in geographic distribution of Beijing strains and the variation in association with drug resistance. However, few studies have distinguished between subgroups of the Beijing clade, or studies were limited in the number of strains analyzed (*8**,**9**,**11**,**12*).

## The Study

We used 3 large data sets from previously described studies to investigate possible differences in correlation with resistance and BCG vaccination between sublineages of the Beijing clade. Details about drug-susceptibility testing, DNA fingerprinting, and demographics by origin can be found elsewhere (5,*13*,*14*). In the Netherlands, 415 (6%) of 6,829 *M. tuberculosis* isolates with available IS*6110* RFLP patterns from 1993 through 2000 were of the Beijing clade (*13*); approximately one third of cases each originated in the Netherlands and Asia and the remaining one third in other areas (*13*). In Vietnam, 301 (53%) of 563 isolates from new TB cases, collected during 1998–1999 mainly in Ho Chi Minh City, belonged to the Beijing clade (*5*). In Hong Kong Special Administrative Region, People’s Republic of China, 355 (71%) of 500 randomly selected *M. tuberculosis* isolates collected during 1998–1999 from patients before treatment were of the Beijing clade (*14*). Information about patient sex and age was available from all 3 sites. Drug susceptibility data and BCG status of patients (presence/absence of BCG scar) were not available from Hong Kong. The patients in this study were treated according to World Health Organization guidelines, independent from their *M. tuberculosis* isolates’ genotype.

**Figure 1:**
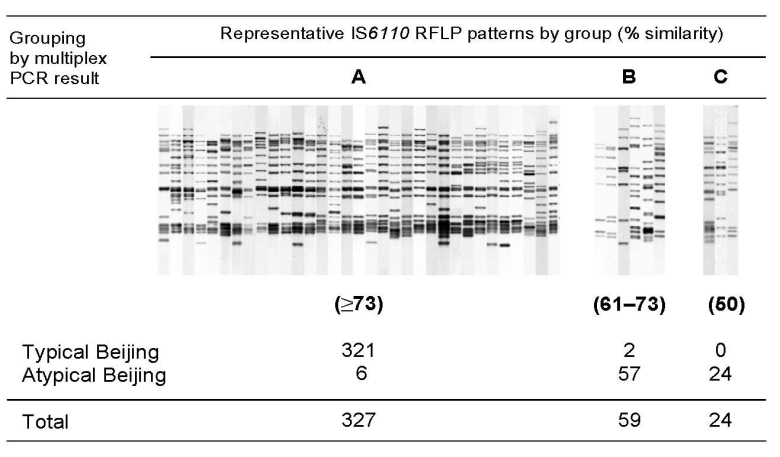
Correlation between the multiplex PCR results and insertion sequence (IS)*6110* restriction fragment length polymorphism (RFLP) pattern similarity. On the basis of IS*6110* RFLP pattern similarity, 3 groups of related patterns (A, B, and C) were recognized among 410 *Mycobacterium tuberculosis* Beijing clade strains isolated in the Netherlands. The similarity of the IS*6110* RFLP patterns of groups B (61%–73%) and C (50%) are relative to the RFLP patterns of group A. Within group C the similarity of the patterns was at least 58%. By using an IS*6110* RFLP similarity of 73% as cutoff (which defined the most homogeneous IS*6110* RFLP group [group A]), 321 (98.2%) of 327 Beijing strains were consistently identified as typical Beijing, and 81 (97.6%) of the 83 remaining Beijing clade strains were classified as atypical Beijing by the multiplex PCR. The κ value for agreement between the tests is 0.922.

Beijing clade strains were defined by their spoligotype pattern (*6*). We used the multiplex PCR of Plikaytis et al. (*15*) to differentiate 3 subgroups of the Beijing clade (W strain, typical, atypical). A specific IS*6110* insertion in the NTF region is detected in typical Beijing strains (*7*). W strains, a subgroup of typical Beijing strains, contain this IS*6110* and an additional IS*6110* insertion in this region (*7*,*15*). Figure 1 shows the correlation between the multiplex PCR results and IS*6110* RFLP similarity.

A total of 1,023 *M. tuberculosis* Beijing clade isolates (410 from the Netherlands, 268 from Vietnam, and 345 from Hong Kong) were available for multiplex PCR analysis. Mean age category of patients was 25–34 years, and >75% were <45 years of age. Because the W strain occurred infrequently, we included it in our analysis of the typical Beijing strain.

**Figure 2:**
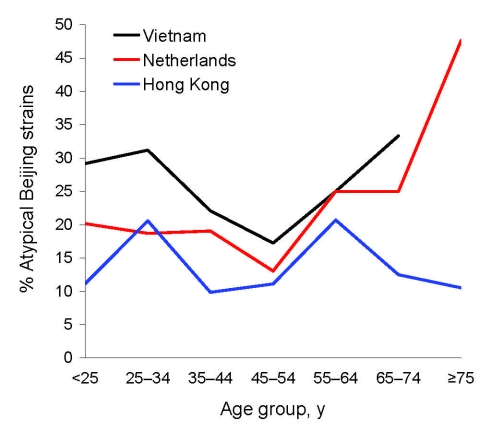
Proportion of atypical Beijing strains among persons with *Mycobacterium tuberculosis* Beijing clade strains in Vietnam, the Netherlands, and Hong Kong, by patient age. The data point of the >75-year age category from the data from Vietnam was omitted because the group contained only 1 patient.

Typical and atypical subgroups were equally distributed among men and women but varied by country and patient age. Atypical Beijing strains occurred in 25.4% (68/268) of isolates in Vietnam, 21.2% (87/410) in the Netherlands, and 13.6% (47/345) in Hong Kong. Atypical Beijing strains were encountered less frequently in Hong Kong than in Vietnam (p<0.001) and the Netherlands (p = 0.007). The Beijing subgroups were equally prevalent among persons of different age groups in Hong Kong, but atypical Beijing strains occurred more frequently in older persons in the Netherlands and Vietnam (Figure 2). This increase in proportion of atypical Beijing strains in older persons was significant in the Netherlands (χ^2^_trend_ 4.5, p<0.035). Combined data for the Netherlands and Vietnam showed significantly more atypical Beijing isolates among patients >75 years of age (odds ratio [OR] 2.96, 95% confidence interval [CI] 1.15–7.67) (Table 1), which suggests more recent introduction and spread of typical Beijing strains.

**Table 1:** Risk factors for atypical *Mycobacterium tuberculosis* in 678 Beijing strain–infected persons with tuberculosis, Vietnam and the Netherlands

Risk factor	Total no. patients infected with Beijing clade	No. (%) patients infected with atypical Beijing strain	Odds ratio (95% confidence interval)	
			Crude	Adjusted*
Country				
Vietnam	268	68 (25.4)	1	1
The Netherlands	410	87 (21.2)	0.79 (0.55–1.14)	0.82 (0.52–1.29)
Sex				
M	415	93 (22.4)	1	1
F	219	48 (21.9)	0.97 (0.66–1.44)	1.01 (0.67–1.51)
Unknown	44	14 (31.8)	1.62 (0.82–3.17)	1.92 (0.90–4.11)
Age, y				
<25	164	36 (22.0)	1	1
25–34	215	51 (23.7)	1.11 (0.68–1.80)	1.05 (0.64–1.73)
35–44	140	29 (20.7)	0.93 (0.54–1.61)	0.80 (0.45–1.42)
45–54	51	7 (13.7)	0.57 (0.24–1.36)	0.45 (0.18–1.12)
55–64	36	9 (25.0)	1.19 (0.51–2.75)	1.03 (0.44–2.45)
65–74	50	13 (26.0)	1.25 (0.60–2.60)	1.07 (0.50–2.30)
>75	22	10 (45.5)	2.96 (1.18–7.41)	2.96 (1.15–7.67)
BCG† vaccination				
No	249	69 (27.7)	1	1
Yes	265	55 (20.8)	0.68 (0.46–1.03)	0.60 (0.38–0.95)
Unknown	164	31 (18.9)	0.61 (0.38–0.98)	0.61 (0.36–1.04)
Total	678	155 (22.8)		

*Adjusted for country, sex, age group, and BCG vaccination. †BCG, *Mycobacterium bovis* bacillus Calmette-Guérin.

To determine whether BCG vaccination might drive this shift in prevalence of the 2 Beijing subgroups, we investigated their distribution in persons vaccinated and not vaccinated with BCG. Of 249 nonvaccinated persons, 27.7% were infected with atypical strains; of 265 vaccinated persons, a significantly lower proportion (20.8%) were infected with atypical strains (adjusted OR 0.60, 95% CI 0.38–0.95) (Table 1). The proportions per genotype emphasized this finding; 44.4% of atypical Beijing strains and 53.8% of typical Beijing strains were isolated from vaccinated persons. The association between typical Beijing strains and vaccination was strong in the data from the Netherlands: 14.2% of Beijing strains isolated from vaccinated persons and 31% of those from nonvaccinated persons were atypical (adjusted OR 0.39, 95% CI 0.20–0.76). In Vietnam, the proportions were nonsignificant (26.2% and 24.4%, respectively).

The unknown BCG vaccination status of 164 of 678 Beijing strain–infected patients is a limitation of our study. In these patients (from the Netherlands), the proportion of atypical Beijing strains was lower and almost similar to that for vaccinated patients. Therefore, if all patients with unknown BCG status were considered nonvaccinated, the association with typical Beijing strains and BCG vaccination would disappear. However, the Netherlands’ National Tuberculosis Register most likely lacks BCG status data because the BCG status for these patients was not checked; we assume the absence of these data introduced no bias. To investigate this further, we extended the analysis of the population in the Netherlands by including all patients with known BCG status (n = 4,004). The proportions of typical Beijing strains for BCG-vaccinated and BCG-nonvaccinated persons were 54.2% and 45.8%, respectively. For all strains other than Beijing strains (i.e., atypical Beijing strains and *M. tuberculosis* strains of all other genotypes), these proportions were 44.5% and 55.5%, respectively. Thus, including all isolates from persons with known BCG status, typical Beijing strains still were isolated significantly more often from BCG-vaccinated than BCG-nonvaccinated persons (p = 0.008). Our findings of significantly fewer typical Beijing isolates among patients >75 years of age (mostly experiencing reactivation and thus representing the population structure of *M. tuberculosis* of decades ago) and significantly more isolates of typical Beijing strains from BCG-vaccinated persons support the hypothesis that BCG vaccination might favor the spread of the typical Beijing strains.

Drug resistance of *M. tuberculosis* Beijing subgroups varied by country (Table 2). In Vietnam, drug resistance was significantly higher than in the Netherlands; 6.7% of Beijing strains in Vietnam compared with 2.0% in the Netherlands were multidrug resistant (MDR), 32.1% compared with 11.0% were isoniazid (INH) resistant, and 44.0% compared with 15.9% were streptomycin resistant (Table 2). Atypical Beijing isolates were more often INH resistant (25.2%) than were typical Beijing isolates (17.6%). Furthermore, atypical Beijing strains were more often MDR (7.1% compared with 2.9%). Atypical Beijing strains were less often streptomycin resistant (21.3% compared with 28.7%) (Table 2). Thus, atypical Beijing isolates were associated with INH resistance and MDR and significantly less likely to be streptomycin resistant than typical Beijing isolates. Similar differences in drug resistance recently were found for the 2 Beijing subgroups among isolates circulating in Japan (*12*) and in the Beijing region of China (*9*), but the findings in China were not statistically significant, probably because of the limited number of stains analyzed. These differences in drug resistance associations suggest the different Beijing sublineages might have different mechanisms of drug resistance development.

**Table 2:** Risk factors for drug resistance of *Mycobacterium tuberculosis* Beijing clade strains in 678 persons with tuberculosis, Vietnam and the Netherlands*

Risk factor	Total no. patients	Resistance, % patients				Adjusted odds ratio (95% confidence interval)†		
		Multidrug	INH	SM		Multidrug	INH	SM
Genotype								
Typical Beijing	523	2.9	17.6	28.7		1	1	1
Atypical Beijing	155	7.1	25.5	21.3		2.48 (1.09-5.65)	1.58 (1.00–2.49)	0.59 (0.37–0.93)
Country								
Vietnam	268	6.7	32.1	44.0		1	1	1
The Netherlands	410	2.0	11.0	15.9		0.21 (0.06–0.76)	0.20 (0.11–0.34)	0.21 (0.13–0.33)
Sex								
M	415	3.6	18.6	28.0		1	1	1
F	219	2.7	19.2	22.4		0.91 (0.34–2.44)	1.34 (0.86–2.11)	0.87 (0.58–1.31)
Unknown	44	11.4	27.3	40.9		2.39 (0.67–8.54)	0.90 (0.41–1.97)	0.88 (0.43–1.78)
Age, y								
<25	164	3.7	13.4	25.0		1	1	1
25–34	215	4.7	24.7	28.4		0.93 (0.32–2.70)	1.76 (1.00–3.12)	0.94 (0.57–1.54)
35–44	140	4.3	25.0	30.0		0.71 (0.21–2.41)	1.46 (0.78–2.74)	0.82 (0.47–1.43)
45–54	51	3.9	17.6	33.3		0.68 (0.12–3.77)	0.91 (0.37–2.27)	0.92 (0.44–1.95)
55–64	36	2.8	16.7	27.8		0.60 (0.07–5.45)	0.99 (0.35–2.81)	1.05 (0.44–2.52)
65–74	50	2.0	10.0	16.0		0.40 (0.04–3.66)	0.63. (022–1.85)	0.57 (0.23–1.39)
>75	22	0	4.5	18.2		--	0.31 (0.04–2.50)	1.06 (0.32–3.49)
BCG vaccination								
No	249	4.0	22.1	28.9		1	1	1
Yes	265	4.2	19.6	30.9		0.63 (0.22–1.83)	0.71 (0.43–1.17)	0.99 (0.63–1.54)
Unknown	164	3.0	14.6	17.7		2.44 (0.54–11.04)	1.64 (0.84–3.21)	1.26 (0.70–2.24)
Total	678	26	131	183				

*INH, isoniazid; SM, streptomycin; BCG, *Mycobacterium bovis* bacillus Calmette-Guérin. †Adjusted for genotype, country, sex, age group, and BCG vaccination.

Despite the association of atypical Beijing strains with INH and multidrug resistance found in this study, typical Beijing strains contribute most substantially to the worldwide MDR TB epidemic (*1**,**4**,**11*). However, in studies showing an association between typical Beijing strains and multidrug resistance, these strains usually also were resistant to streptomycin (as we also found). Typical Beijing strains may therefore become streptomycin resistant more easily, eventually leading to MDR TB, as the W-strain outbreak in New York showed (*16*). Alternatively, the increased prevalence of typical Beijing strains in the current global *M. tuberculosis* population may be caused not by drug-driven selection but by their hypervirulence (*2*), higher adaptability (*10*), higher rate of progression to disease, greater ability to circumvent BCG-induced immunity (*2,3*, this study), or other specific features.

## Conclusions

We showed that subgroups of the *M. tuberculosis* Beijing clade have different associations with drug resistance and BCG vaccination. Individual lineages of the Beijing clade are likely to be evolving in different areas, possibly because of intrinsic strain characteristics, differences in anti-TB drug regimens and BCG-vaccination strategies in different areas, chance, or a combination of these. Thus, anti-TB drugs and BCG vaccination influence the dynamics in the population structure of *M. tuberculosis*. The efficacy of new candidate TB vaccines therefore should be tested against a broad panel of epidemic strains from all high-prevalence areas (*4*). Furthermore, treatment of infections by different *M. tuberculosis* genotypes might require different anti-TB treatment strategies. More extended studies are needed in high-prevalence settings, especially studies of other predominant genotype families of *M. tuberculosis*.
